# Neuropsychiatric complications and associated management in adolescent and young adult cancer survivors: An *All of Us* study

**DOI:** 10.1002/cam4.6641

**Published:** 2023-10-30

**Authors:** Ivann Agapito, Ding Quan Ng, Joel Milam, Argyrios Ziogas, Hoda Anton‐Culver, Alexandre Chan

**Affiliations:** ^1^ School of Pharmacy and Pharmaceutical Sciences University of California Irvine Irvine California USA; ^2^ School of Medicine University of California Irvine Irvine California USA; ^3^ Program in Public Health University of California Irvine Irvine California USA

**Keywords:** adolescent and young adult cancer, *All of Us*, matched controls, neuropsychiatric, past medical history, survivor

## Abstract

**Background:**

About 4.5% of new cancer cases affect adolescent and young adult aged between 15 and 39 years in the United States (US). However, the effect of neuropsychiatric conditions on long‐term adolescent and young adult cancer (AYAC) survivors has not been formally investigated. Thus, the impact and management of late neuropsychiatric complications in AYAC survivors compared to non‐cancer‐matched controls (NCMC) in the US were evaluated using the *All of Us* (*AoU*) Research Program.

**Methods:**

Participants in the *AoU* Controlled Tier Dataset (v6) diagnosed with cancer between ages 15 and 39 were identified from electronic health records and surveys. AYAC survivors were matched with NCMC using the optimal pair‐matching algorithm at a 1:4 ratio. Data on past diagnoses, current follow‐up care, and treatment patterns of neuropsychiatric complications were collected.

**Results:**

Analysis was performed on 788 AYAC survivors and 3152 NCMC. AYAC survivors, with an average of 8.8 years since their first cancer diagnosis, were more likely than NCMC to receive a diagnosis of neuropathy, memory loss and epilepsy (*p*  < 0.001). Survivors also had a higher rate of follow‐up care and treatment utilization for these neurological conditions compared to NCMC (*p*  < 0.05). Treatment utilization was highest among survivors receiving care for epilepsy (88%), and lower for neuropathy (70%), memory loss (61%), and chronic fatigue (59%).

**Conclusions:**

This large study reveals that AYAC survivors, on average 9 years after their cancer diagnosis, require more frequent follow‐up care for neurological complications compared to non‐cancer individuals. However, the management of neuropathy, memory loss, and chronic fatigue is hindered by a lack of mechanism‐based effective therapies.

## 1 BACKGROUND

An adolescent or young adult cancer (AYAC) survivor is an individual 15–39 years of age at the initial cancer diagnosis.^1^ Annually, over 87,000 AYACs in the United States (US) are diagnosed with cancer, constituting 4.5% of all new cancer cases. AYAC survivors are distinct from younger/older cancer patients and suffer from delays in diagnosis, limited access to appropriate treatment, low adherence to therapy, low clinical trial enrollment, treatment‐related toxicity, and unique psychosocial challenges.^2^, ^3^ Posttreatment health issues among AYAC survivors are becoming increasingly relevant, and more in‐depth research is needed within this category of patients.^4^, ^5^, ^6^, ^7^ For these reasons, the National Cancer Institute (NCI) has declared AYAC patients as a vulnerable population.^2^

Adolescent or young adult cancer survivors often experience a myriad of treatment‐related chronic and late toxicities that can lead to functional impairments at high economic, emotional, and social costs.^4^ Several studies described long‐term complications in AYAC survivors. However, many of these single‐institution or single‐disease cohorts are small in sample size and lack appropriate controls, due to the rarity of cancer diagnosis among AYAC survivors.^8^, ^9^ Furthermore, these studies rarely evaluate the long‐term follow‐up and management of these conditions.^10^, ^11^

Literature on long‐term complications has shown significant and persistent associations of neuropathy, depression, fatigue, insomnia, and cognitive toxicity among pediatric, adult, and older patients with breast cancer and other cancer types.^12^, ^13^, ^14^, ^15^ These neuropsychiatric complications stand out due to gaps in research and practice among central and peripheral nervous system assessment, diagnosis, and management in AYAC survivorship. Hence, we designed a study to assess the prevalence and long‐term management of neuropsychiatric complications in AYAC survivors compared to non‐cancer‐matched controls (NCMC) using the *All of Us* (*AoU*) Research Program. *AoU* is managed by the National Institutes of Health (NIH) to promote research among a diverse set of participants to advance precision medicine and uncover new insights into human health.^16^ At the time of this analysis, the program has recruited over 300,000 participants from across the US, providing a wealth of data involving health surveys and electronic health records (EHR) useful to study niche populations that are underrepresented in research.^16^ Using a hypothesis‐generating approach, we examined a broad range of neuropsychiatric diagnoses self‐reported in the *AoU* program. Findings from this study will provide important insights to clinicians and researchers on the management of neuropsychiatric conditions that must be prioritized for follow‐up among AYAC survivors.

## 2 METHODS

### 2.1 Study design and data sources

This study is a secondary data analysis of a US nationwide prospective cohort study, the *AoU* Research Program. The program aims to recruit 1 million participants ≥18 years old across 340 recruitment sites. Recruitment began in May 2018 and is ongoing. All consented participants complete three baseline surveys (Basics, Overall Health, and Lifestyle) and have the option to complete the “Personal Medical History” (PMH) survey, uploaded on https://www.researchallofus.org/data‐tools/survey‐explorer. EHR data are mined, and all data, including those from surveys, are organized in the Observational Health and Medicines Outcomes Partnership (OMOP) common data model v5.2.^17^

### 2.2 Population

Eligible participants are required to complete cancer and neuropsychiatric conditions sections of the PMH surveys to provide data for analysis in our study. We identified two cohorts of participants: AYAC survivors and NCMC. **AYAC survivors** were selected if they had a registered EHR record for cancer between ages 15 and 39 years old, further confirmed with PMH survey responses, and were ≥1 year(s) from their first cancer diagnosis. Survivors greater than 40 years old at time of survey could be eligible if they received their first cancer diagnosis between 15 and 39 years old. The study included AYAC who were at least 1 year post‐diagnosis, as most would have completed primary treatment and entered survivorship within the first year in the US.^18^ ICD‐9‐CM (140–209) and ICD‐10‐CM (C00–C96) codes were identified using the *AoU* concept set‐building dashboard to identify cancer diagnoses in the EHR.

**FIGURE 1:**
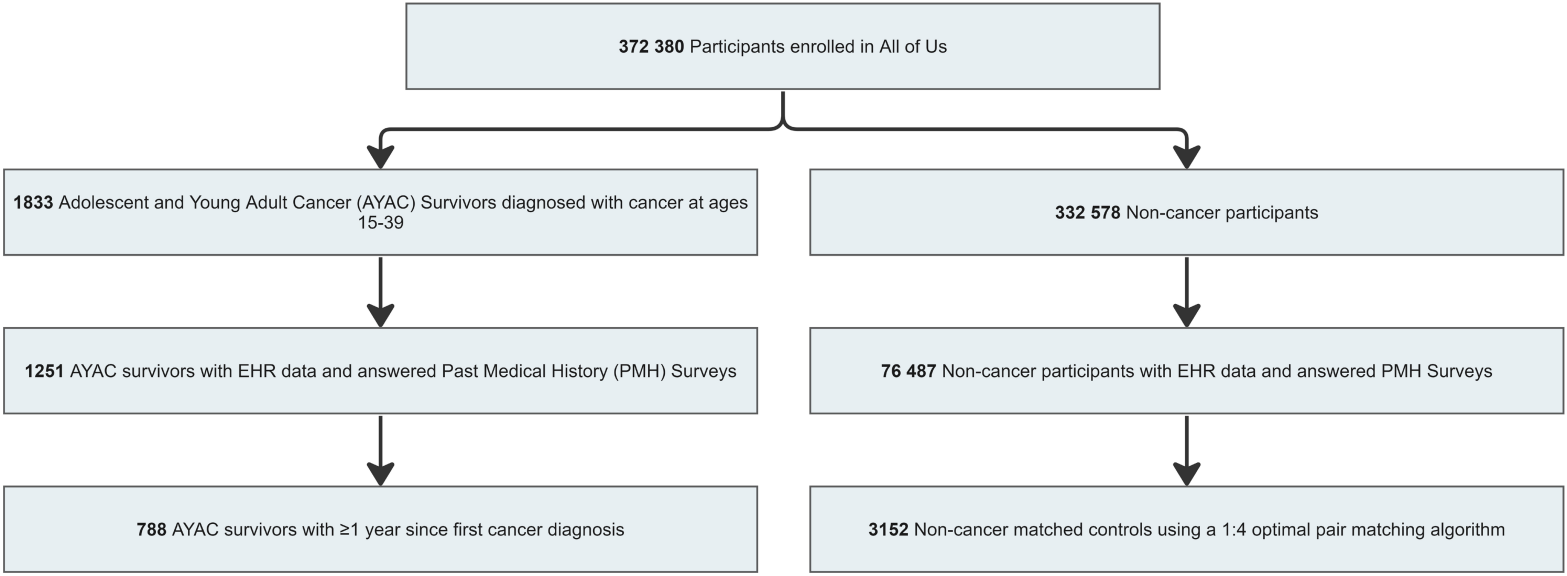
Selection of adolescent and young adult cancer survivors and non‐cancer matched controls. AYAC survivors were selected from the *All of Us* (*AoU*) database using age at first cancer diagnosis and ICD‐9‐CM and ICD‐10‐CM codes from electronic health record (EHR) data. AYAC survivors were verified of their cancer conditions using past medical history (PMH) surveys. NCMC were propensity‐score matched using a 1:4 optimal pair matching algorithm with the following matching parameters: sex at birth, gender, race, ethnicity, highest education level, annual household income, and age at survey completion.

The **NCMC cohort** included participants without prior cancer diagnoses listed in either the EHR or PMH survey. AYAC survivors were propensity‐score matched with NCMC in a 1:4 ratio using the optimal pair‐matching algorithm.^19^, ^20^ The ratio was selected for enhancing the precision of effect sizes without overfitting.^21^ Matching parameters included sex at birth, gender, race, ethnicity, highest education level, annual household income, and age at survey completion. (Figure 1).

### 2.3 Covariates

Sociodemographic information on sex at birth, gender, race, ethnicity, highest education level, and annual household income was accessed from participants' responses to the “Basics” survey. We calculated the age at survey by leveraging the participants' birth and survey completion dates. The age of first cancer diagnosis was determined using the first record of a cancer diagnosis in the EHR. Years from first cancer diagnosis were defined as the length of time, in years, between the dates of first cancer diagnosis and survey completion. The site of cancer for AYAC survivors was determined using the PMH survey.

### 2.4 Outcomes

A total of 16 neurologic conditions and 12 psychiatric conditions were identified using the PMH survey results provided and predetermined by the *AoU* program.^22^ The primary outcomes are the odds of receiving a past diagnosis (*Has a doctor or health care provider ever told you that you have…?*) for a specific neuropsychiatric condition determined by comparing AYAC survivors against NCMC (reference group). Secondary outcomes include the proportions of receiving a past diagnosis, the odds and proportions of seeing a provider (*Are you still seeing a doctor or health care provider for…?*), and the odds and proportions of receiving ongoing medications or treatment (*Are you currently prescribed medications and/or receiving treatment for…?*) for neuropsychiatric conditions at the time of the survey, comparing between AYAC survivors and NCMC.

### 2.5 Statistical analysis

Complete case analysis was performed. We summarized continuous variables using means, ranges, standard deviations (SD), and categorical variables with counts and percentages. In addition, we reported results in compliance with the *AoU* Data and Statistics Dissemination Policy prohibiting the display of participant counts ranging from 1 to 20. The standardized mean differences (SMDs) after matching were analyzed to evaluate the success of the matching algorithm.^23^ Employing a doubly robust methodology, inferential analyses were performed with multiple logistic regression, adjusting for covariates (sex at birth, gender, race, ethnicity, highest education level, annual household income, and age at survey completion) to determine the associations between cancer diagnoses and neuropsychiatric complications. We then presented effect sizes as adjusted odds ratios (AOR) and 95% confidence intervals (CI). Subgroup analysis, stratified based on years since first diagnosis (1–5, 6–10, >10 years), was performed to evaluate the persistence and long‐term management of these symptoms. All statistical tests were two‐sided. For the primary outcome, multiple testing was accounted, with a Bonferroni‐corrected significance level of 0.00179 to correct for the number of neuropsychiatric complications (28 in total) evaluated as part of the primary outcome. Other *p* values were set at 0.05 for statistical significance. Data were accessed with Google BigQuery and analyzed using R v4.1.2 in an integrated Jupyter Notebook environment, and matching was completed with R package MatchIt (4.4.0).^24^, ^25^

## 3 RESULTS

### 3.1 Descriptive statistics

We accessed the *AoU* Controlled Tier Dataset version 6 (C2022Q2R2), and data were current as of January 1, 2022. *AoU* recruited 372,380 participants, of which 142,090 consented to provide EHR data and completed the PMH surveys. Of these, 788 participants met our eligibility criteria for AYAC survivors and matched them to 3152 NCMC (Figure 1). At the time of the survey, the mean age was 41.3 years, and AYAC survivors averaged 8.8 (SD = 8.2) years from their initial cancer diagnoses. There were higher proportions of female (75.5% vs 73.0%) and White (87.9% vs 86.1%) participants among AYAC survivors compared to NCMC (Table 1). Nevertheless, the groups were well‐matched as the SMDs for all matched variables achieved the threshold of <0.1. The most common cancers among AYAC survivors were skin (21.3%), breast (18.5%), and thyroid cancers (16.9%) (Table 1).

**TABLE 1:** Descriptive statistics of adolescent and young adult cancer survivors and non‐cancer‐matched controls cohorts.

Demographic variables	AYAC^a^ (*N* = 788)	NCMC^a^ (*N* = 3152)	SMD
Mean age when surveyed (range, SD)	41.3 (20–75, 9.7)	41.4 (18–84)	0.003
Mean years since first cancer diagnosis (range, SD)	8.8 (1–40, 8.2)	–	–
Mean age at first cancer diagnosis (range, SD)	32.5 (15–39, 5.5)	–	–
Gender, *n* (%)			
Female	595 (75.5)	2302 (73.0)	0.058
Male	182 (23.1)	774 (24.6)	0.035
Sex at birth, *n* (%)			
Female	602 (76.4)	2356 (74.8)	0.039
Male	186 (23.6)	796 (25.3)	0.039
Race, *n* (%)			
White	693 (87.9)	2714 (86.1)	0.057
Black or African American	44 (5.6)	200 (6.3)	0.033
Asian	24 (3.0)	110 (3.5)	0.026
More than one population	≤20	104 (3.3)	0.068
Middle Eastern or North African	≤20	≤20	0.038
Native Hawaiian or Other Pacific Islander	≤20	≤20	0.000
Ethnicity, *n* (%)			
Not Hispanic or Latino	763 (96.8)	3033 (96.2)	0.034
Hispanic or Latino	25 (3.2)	119 (3.8)	0.034
Education, *n* (%)			
≤High school graduate or GED	67 (8.5)	330 (10.5)	<0.100
College one to 3 years	168 (21.3)	758 (24.0)	0.067
College graduate	275 (34.9)	1043 (33.1)	0.038
Advanced degree	278 (35.3)	1021 (32.4)	0.060
Income, *n* (%)			
<$10 k	35 (4.4)	159 (5.0)	0.029
$10 k–$25 k	72 (9.1)	288 (9.1)	0.000
$25 k–$35 k	48 (6.1)	225 (7.1)	0.044
$35 k–$50 k	66 (8.4)	281 (8.9)	0.019
$50 k–$75 k	111 (14.1)	467 (14.8)	0.021
$75 k–$100 k	105 (13.3)	453 (14.4)	0.031
$100 k–$150 k	156 (19.8)	608 (19.3)	0.013
$150 k–$200 k	73 (9.3)	277 (8.8)	0.016
>$200 k	122 (15.5)	394 (12.5)	0.082
Cancer conditions, *n* (%)			
Blood	88 (11.2)	–	–
Bone	24 (3.0)	–	–
Brain	53 (6.7)	–	–
Breast	146 (18.5)	–	–
Cervical	42 (5.3)	–	–
Colorectal	26 (3.3)	–	–
Kidney	24 (3.0)	–	–
Ovarian	24 (3.0)	–	–
Other^b^	173 (22.0)	–	–
Skin	168 (21.3)	–	–
Thyroid	133 (16.9)	–	–

Abbreviations: AYAC, adolescent and young adult cancer; GED, tests of general educational development; NCMC, non‐cancer matched controls; SD, standard deviation; SMD, standardized mean difference.

^a^ Results reported in compliance with the *All of Us* Data and Statistics Dissemination Policy prohibiting the display of participant counts ranging 1‐20.

^b^ Cumulation of head/neck, endocrine, endometrial, lung, stomach, bladder, eye, pancreatic, prostate, and esophageal cancers.

AYAC survivors who did not answer the PMH survey (*n* = 785) comprised of fewer White participants, achieved a lower education level, and had lower household income. This data were summarized in Table S1.

### 3.2 Past diagnoses of neuropsychiatric condition

The most observed psychiatric conditions among AYAC were depression (38.1%), anxiety (36.2%), and post‐traumatic stress disorder (12.9%), whereas the most commonly observed neurological conditions were migraine (27.7%), neuropathy (13.8%), and insomnia (12.3%) (Tables 2 and 3). Bivariate analysis revealed statistically higher proportions of migraine, neuropathy, chronic fatigue, memory loss, and restless leg syndrome but lower proportions of ADHD among AYAC survivors compared to NCMC (*p* < 0.05). Although the prevalence was similar for psychiatric conditions between AYAC and NCMC (Table 2), AYAC were more likely to be diagnosed with depression and bipolar disorder than NCMC between 18 and 64 years old (*p* < 0.05, Table S2). After controlling for differences in covariates, AYAC survivors were more likely to report a past diagnosis of neuropathy (AOR = 3.79, 95% CI = 2.89–4.98, *p* < 0.001), chronic fatigue (AOR = 1.76, 95% CI = 1.31–2.35, *p* < 0.001), memory loss (AOR = 2.79, 95% CI = 1.95–4.01, *p* < 0.001), and epilepsy (AOR = 2.46, 95% CI = 1.67–3.62, *p* < 0.001; Tables 2 and 3).

**TABLE 2:** Proportions and adjusted odds of self‐reporting past diagnoses of psychiatric conditions among adolescent and young adult cancer survivors compared to non‐cancer‐matched controls.

Psychiatric conditions	AYAC^a^ (*N* = 788)	NCMC^a^ (*N* = 3152)	*p* ^‡^ Value	AOR^b^ (95%CI)	*p* ^§^ Value
ADHD, *n* (%)	69 (8.2)	359 (11.4)	0.034^c^	0.79 (0.60–1.04)	0.099
Alcohol disorder, *n* (%)	≤20	107 (3.3)	0.075	0.65 (0.39–1.11)	0.113
Anxiety, *n* (%)	277 (35.2)	1100 (34.9)	0.894	1.05 (0.89–1.25)	0.547
Autism, *n* (%)	≤20	46 (1.5)	0.337	0.84 (0.38–1.84)	0.655
Bipolar disorder, *n* (%)	33 (4.2)	176 (5.6)	0.118	0.81 (0.55–1.20)	0.289
Depression, *n* (%)	300 (38.1)	1253 (39.8)	0.388	0.95 (0.81–1.14)	0.653
Drug‐use disorder, *n* (%)	≤20	87 (2.8)	0.345	0.81 (0.47–1.40)	0.456
Eating disorder, *n* (%)	38 (4.8)	168 (5.3)	0.567	0.92 (0.64–1.33)	0.648
Personality disorder, *n* (%)	≤20	65 (2.1)	0.778	1.06 (0.59–1.91)	0.833
PTSD, *n* (%)	102 (12.9)	350 (11.1)	0.147	1.28 (1.01–1.63)	0.045
Schizophrenia, *n* (%)	≤20	≤20	0.408	1.92 (0.63–5.79)	0.249
Social phobia, *n* (%)	29 (3.7)	105 (3.3)	0.629	1.23 (0.80–1.90)	0.348

Abbreviations: ADHD, attention‐deficit/hyperactivity disorder; AYAC, adolescent and young adult cancer; AOR, adjusted odds ratio; CI, confidence interval; NCMC, non‐cancer matched controls; *p* ^‡^, *p* values for Pearson's chi‐square test; *p* ^§^, *p* values for multiple logistic regression; PTSD, post‐traumatic stress disorder.

^a^ Results were reported in compliance with the *All of Us* Data and Statistics Dissemination Policy prohibiting the display of participant counts ranging 1–20.

^b^ Adjusted for sex at birth, gender, race, ethnicity, highest education level, annual household income, and age at survey completion. NCMCs served as the reference group.

^c^ *p* < 0.05 for *p* ^‡^.

**TABLE 3:** Proportions and adjusted odds of self‐reporting past diagnoses of neurological conditions among adolescent and young adult cancer survivors compared to non‐cancer‐matched controls.

Neurological conditions	AYAC^a^ (*N* = 788)	NCMC^a^ (*N* = 3152)	*p* ^‡^ Value	AOR^b^ (95% CI)	*p* ^§^ Value
Cerebral palsy, *n* (%)	≤20	≤20	1.000	1.02 (0.11–9.61)	0.990
Chronic fatigue, *n* (%)	72 (9.1)	182 (5.8)	<0.001*	1.76 (1.31–2.35)	<0.001**
Concussion, *n* (%)	65 (8.2)	272 (8.6)	0.733	0.94 (0.71–1.25)	0.661
Dementia, *n* (%)	≤20	≤20	0.133	5.25 (0.67–41.08)	0.110
Epilepsy, *n* (%)	44 (5.6)	77 (2.4)	<0.001*	2.46 (1.67–3.62)	<0.001**
Insomnia, *n* (%)	97 (12.3)	313 (9.9)	0.050	1.33 (1.04–1.71)	0.024
Memory loss, *n* (%)	53 (6.7)	87 (2.8)	<0.001*	2.79 (1.95–4.01)	<0.001**
Migraine, *n* (%)	218 (27.7)	724 (23.0)	0.006*	1.29 (1.08–1.55)	0.006
Multiple sclerosis, *n* (%)	≤20	28 (0.9)	0.730	0.83 (0.34–2.03)	0.683
Muscular dystrophy, *n* (%)	≤20	≤20	0.570	1.60 (0.30–8.41)	0.580
Narcolepsy, *n* (%)	≤20	≤20	0.316	1.61 (0.66–3.88)	0.293
Neuropathy, *n* (%)	109 (13.8)	142 (4.5)	<0.001*	3.79 (2.89–4.98)	<0.001**
Parkinson's disease, *n* (%)	≤20	≤20	0.838	1.64 (0.16–16.88)	0.676
Restless leg syndrome, *n* (%)	55 (7.0)	154 (4.9)	0.019*	1.60 (1.15–2.22)	0.005
Spinal cord injury, *n* (%)	≤20	48 (1.5)	0.085	1.86 (1.07–3.23)	0.027
Traumatic brain injury, *n* (%)	≤20	52 (1.6)	0.228	1.48 (0.85–2.56)	0.166

Abbreviations: AYAC, Adolescent and young adult cancer; AOR, adjusted odds ratio; CI, confidence interval; NCMC, non‐cancer matched controls *p* ^‡^, *p* values for Pearson's chi‐square test; *p* ^§^, *p* values for multiple logistic regression.

^a^ Results were reported in compliance with the *All of Us* Data and Statistics Dissemination Policy prohibiting the display of participant counts ranging 1–20.

^b^ Adjusted for sex at birth, gender, race, ethnicity, highest education level, annual household income, and age at survey completion. NCMCs served as the reference group.

* *p* < 0.05 for *p* ^‡^.

** Bonferroni‐corrected *p* < 0.00179 for *p* ^§^.

### 3.3 Currently seeing a provider for neuropsychiatric conditions

Among the 28 neuropsychiatric conditions, more AYAC survivors were experiencing and seeing a provider for neuropathy, chronic fatigue, memory loss, and epilepsy at the time of the survey (*p* < 0.05, Table 4) compared to NCMC. After confounder adjustments, AYAC survivors remained more likely to see a provider for neuropathy (AOR = 2.60, 95% CI = 1.86–3.62, *p* < 0.001), chronic fatigue (AOR = 1.63, 95% CI = 1.14–2.33, *p* = 0.007), memory loss (AOR = 3.41, 95% CI = 2.13–5.46, *p* < 0.001), and epilepsy (AOR = 3.46, 95% CI = 2.15–5.58, *p* < 0.001) compared to NCMC at the time of survey (Table 4).

**TABLE 4:** Adjusted odds of seeing a provider and taking medications/receiving treatment for neuropsychiatric conditions in adolescent and young adult cancer survivors compared to non‐cancer‐matched controls, stratified by years since cancer diagnosis.

Neurological Conditions	Seeing a provider		Taking medications/receiving treatment	
	AOR^a^ (95%CI)	*p* Value	AOR^a^ (95%CI)	*p* Value
Neuropathy				
Years since cancer diagnosis				
Overall	2.60 (1.86–3.62)	<0.001^b^	2.24 (1.53–3.28)	<0.001^b^
1–5 years	3.68 (2.23–6.05)	<0.001^b^	3.76 (2.14–6.62)	<0.001^b^
6–10 years	3.25 (1.44–7.35)	0.005^b^	2.86 (1.15–7.14)	0.024^b^
>10 years	1.61 (0.87–2.96)	0.129	0.89 (0.40–1.97)	0.770
Chronic fatigue				
Years since cancer diagnosis				
Overall	1.63 (1.14–2.33)	0.007^b^	1.64 (1.04–2.59)	0.034^b^
1–5 years	1.97 (1.23–3.18)	0.005^b^	1.83 (0.98–3.41)	0.057
6–10 years	1.02 (0.34–3.08)	0.972	1.70 (0.39–7.49)	0.481
>10 years	1.29 (0.65–2.57)	0.470	1.41 (0.60–3.31)	0.428
Memory loss				
Years since cancer diagnosis				
Overall	3.41 (2.13–5.46)	<0.001^b^	4.17 (2.22–7.83)	<0.001^b^
1–5 years	5.09 (2.43–10.66)	<0.001^b^	5.27 (1.99–13.98)	<0.001^b^
6–10 years	5.33 (1.85–15.34)	0.002^b^	20.92 (1.88–232.45)	0.013^b^
>10 years	1.29 (0.53–3.16)	0.580	1.79 (0.62–5.19)	0.282
Epilepsy				
Years since cancer diagnosis				
Overall	3.46 (2.15–5.58)	<0.001^b^	3.48 (2.10–5.78)	<0.001^b^
1–5 years	3.47 (1.84–6.56)	<0.001^b^	3.23 (1.62–6.44)	<0.001^b^
6–10 years	8.26 (2.41–28.27)	<0.001^b^	10.35 (3.07–34.83)	<0.001^b^
>10 years	3.17 (1.03–9.73)	0.043^b^	2.63 (0.72–9.60)	0.140

Abbreviations: AYAC, Adolescent and young adult cancer; AOR, adjusted odds ratio; CI, confidence interval; NCMC, non‐cancer matched control.

^a^ Adjusted for sex at birth, gender, race, ethnicity, highest education level, annual household income, and age at survey completion. NCMCs served as the reference group.

^b^ *p* < 0.05.

### 3.4 Currently taking medications and/or receiving treatment for neuropsychiatric conditions

After covariate adjustments, AYAC survivors were more likely to report current treatment or receiving medications for neuropathy (AOR = 2.24, 95% CI = 1.53–3.28, *p* < 0.001), chronic fatigue (AOR = 1.64, 95% Cl = 1.04–2.59, *p* = 0.034), memory loss (AOR = 4.17, 95% CI = 2.22–7.83, *p* < 0.001), and epilepsy (AOR = 3.48, 95% CI = 2.10–5.78, *p* < 0.001) at the time of survey compared to NCMC (Table 4).

Among AYAC survivors still seeing a provider for neuropathy (*n* = 61), 43 (70.5%) were taking medications or receiving treatment for neuropathy. The proportions for other neurological conditions are as follows: chronic fatigue (58.7%), memory loss (61.0%), and epilepsy (87.9%; Table S3).

### 3.5 Subgroup analysis

Among 788 AYAC participants, there were 377 (47.8%) with 1–5 years, 167 (21.2%) with 6–10 years, and 244 (31.0%) reporting more than 10 years since cancer diagnosis. Except for chronic fatigue, AYAC participants had higher odds of seeing a provider and receiving treatment for neuropathy, memory loss, and epilepsy up to 10 years since cancer diagnosis (*p* < 0.05, Table 4).

## 4 DISCUSSION

In this large national cohort study of AYAC survivors averaging 9 years post‐diagnosis, we have observed that long‐term AYAC survivors are still seeking care from providers and receiving treatment for neuropathy, chronic fatigue, epilepsy, and memory loss that could be related to their cancer and/or associated treatment. Epilepsy, for example, is frequently experienced by survivors of brain tumors as well as those with brain metastases, and these patients require routine follow‐up care to monitor for recurrent seizures.^26^ On the other hand, chronic fatigue and memory impairment in cancer survivors are often linked to “sickness behavior” characterized by upregulation in pro‐inflammatory cytokines.^27^, ^28^, ^29^ Memory loss and impairments in other cognitive domains (processing speed, executive function, and attention) have been reported in cancer survivors even prior to receiving cancer treatment,^30^ and they could be worsened posttreatment with radiotherapy and chemotherapy.^31^ Neuropathy experienced by survivors often manifests with numbness, tingling, and pain, and these symptoms are linked to the receipt of neurotoxic antineoplastics such as taxanes and platinum agents.^32^ Our findings are important because these complications are not fully reversible years after cancer diagnosis, an observation further validated by our findings of AYAC survivors who averaged 9 years from first cancer diagnosis. These longstanding complications can create physical and emotional burdens to AYAC survivors who are eager to return to normal life and seek to return to normalcy post‐cancer treatment.

Our findings from this sizeable US‐based study have significant implications in the care of AYAC survivors. We recommend that care pathways of AYAC survivors should include routine surveillance for neuropathy, chronic fatigue, epilepsy, and memory loss. Management of these complications remains relevant in the first 10 years of diagnosis as they are known to affect health‐related quality of life.^33^ In the Adolescent and Young Adult Health Outcomes and Patient Experience (AYA HOPE) study, more than 40% and 53% of AYAC patients reported problems with “forgetting” at 6–14 months and 15–35 months after a cancer diagnosis, respectively, with one‐third of patients finding it difficult to pay attention at work or school after a cancer diagnosis.^34^ Fatigue may also affect survivors' work outcomes. Several studies observed a negative association between fatigue symptoms' severity and workability and status.^33^ However, long‐term rehabilitation is often associated with increased medical expenses^35^ and AYAC survivors may not adhere to treatment to avoid financial hardship. The higher medication and treatment utilization for epilepsy may be attributed to clinicians' perception of a more pronounced severity of the complication compared to neuropathy, memory loss, and chronic fatigue. In contrast to a myriad of evidence‐based treatments available for epilepsy and seizures,^36^, ^37^, ^38^ fewer evidence‐based treatment options are shown to be effective for managing neuropathy,^39^ chronic fatigue,^28^ and memory loss,^31^ likely due to the poor understanding of the underlying mechanisms for these complications. Mechanism‐based interventional strategies are urgently needed for these complications that lack effective therapies.

This analysis relies on a database containing secondary data and PMH surveys to verify cancer diagnoses and examine neuropsychiatric outcomes. By doing so, risks of self‐reporting and misclassification biases can occur. In addition, the PMH survey is optional for completion among *AoU* participants; hence, missing data are highly prevalent considering the larger *AoU* cohort of over 300,000 individuals. White participants were also more likely to complete PMH surveys than other racial and ethnic minorities. Specific treatment‐related data for chemotherapies (i.e., chemotherapy, radiotherapy, and surgical resection) is not available in the EHR for all participants in the *AoU* program; thus, the association between treatment and late neuropsychiatric complications within this cohort is limited to PMH surveys. Furthermore, we were unable to establish the temporal relationship between the conditions using the PMH survey. Nevertheless, the higher prevalence of epilepsy, neuropathy, memory loss, and chronic fatigue among AYAC compared to NCMC, together with the vast literature illustrating the characteristics and mechanisms of such complications, have cross‐validated the likelihood that these neurological conditions are key consequences of cancer and the receipt of antineoplastics.

To confirm whether our identified AYAC cohort is generalizable to the US population, we compared the distribution of cancer conditions in our nested cohort against data provided by the Surveillance, Epidemiology, and End Results (SEER) Program. We found similarities between the *AoU* AYAC cohort and the US AYAC population except in male‐related malignancies. It may appear that our sample was skewed toward more females, thus limiting the representation of male AYAC survivors in this study. We did not limit the diagnosis date range; hence, changing paradigm of cancer treatment over time may influence the experience of different late effects. Lastly, the management of AYAC survivors is highly dependent on cancer diagnosis and subtypes; hence, this study did not investigate the associations between neuropsychiatric conditions with specific cancer phenotypes or treatments.

Despite these limitations, our sample size remains large compared to other published studies, providing adequate power to identify associations with multivariate analyses. We also approached the study with a hypothesis‐generating objective that is achieved by examining a broad range of neuropsychiatric conditions self‐reported in the survey. Future studies may utilize EHR or claims data to validate our prevalence findings. Finally, our study is innovative because our AYAC survivors averaged 9 years post‐diagnosis, providing unique data for neuropathy, fatigue, memory loss, and epilepsy as potential cancer‐related neurological complications that continue years after curative treatment. Importantly, our findings have revealed key unmet needs in the management of these complications and set the groundwork necessary to investigate causal pathways for developing interventions to ameliorate these conditions.

## 5 CONCLUSION

By employing the *AoU* Research Program, a US nationwide prospective cohort of adult individuals, we observed higher odds of follow‐up care and treatment for epilepsy, neuropathy, memory loss, and chronic fatigue among AYAC survivors who were 9 years post‐cancer diagnosis compared to NCMC. The occurrence and persistence of these complications during and after receiving a cancer diagnosis at ages 15–39 can negatively hinder their transition across these critical life stages of completing higher education, family building, and work progression. Our findings support the urgency in addressing the unmet needs regarding the lack of effective therapies providers can recommend for managing neurological complications during survivorship care. We urge researchers to develop mechanism‐based interventional strategies for these complications in this NCI‐designated vulnerable population of patients and survivors.

## AUTHOR CONTRIBUTIONS

**Ivann Agapito:** Conceptualization (equal); data curation (equal); formal analysis (equal); methodology (equal); writing – original draft (equal); writing – review and editing (equal). **Ding Quan Ng:** Conceptualization (equal); data curation (equal); formal analysis (equal); methodology (equal); writing – original draft (equal); writing – review and editing (equal). **Joel Milam:** Writing – review and editing (equal). **Argyrios Ziogas:** Writing – review and editing (equal). **Hoda Anton‐Culver:** Writing – review and editing (equal). **Alexandre Chan:** Conceptualization (equal); formal analysis (equal); methodology (equal); supervision (lead); writing – original draft (equal); writing – review and editing (equal).

## CONFLICT OF INTEREST STATEMENT

The authors declare no potential conflict of interests with this research.

## ETHICAL STATEMENT

The study conducted in this research has undergone the exempt self‐determination process at the University of California Irvine Institutional Review Board (IRB). Ethical approval was not required or sought as it falls under the Category 4 exemption of IRB review. The authors of the study did not directly communicate with the participants, and all potentially identifying information has been removed from the data available in the *AoU* Researcher Workbench. To gain access to the research data, the authors completed necessary research ethics training administered by the *AoU* Research Program and must adhere to the *AoU* Data User Code of Conduct for upholding data privacy and confidentiality.

## ACKNOWLEDGEMENTS

The *All of Us* Research Program is supported by the National Institutes of Health, Office of the Director: Regional Medical Centers: 1 OT2 OD026549; 1 OT2 OD026554; 1 OT2 OD026557; 1 OT2 OD026556; 1 OT2 OD026550; 1 OT2 OD 026552; 1 OT2 OD026553; 1 OT2 OD026548; 1 OT2 OD026551; 1 OT2 OD026555; IAA #: AOD 16037; Federally Qualified Health Centers: HHSN 263201600085U; Data and Research Center: 5 U2C OD023196; Biobank: 1 U24 OD023121; The Participant Center: U24 OD023176; Participant Technology Systems Center: 1 U24 OD023163; Communications and Engagement: 3 OT2 OD023205; 3 OT2 OD023206; and Community Partners: 1 OT2 OD025277; 3 OT2 OD025315; 1 OT2 OD025337; 1 OT2 OD025276. In addition, the *All of Us* Research Program would not be possible without the partnership of its participants.

## Supporting information


Tables S1–S3.

## Data Availability

Data used in this research is available for reproduction under the *All of Us* Research database at https://www.researchallofus.org/.

## References

[1] Coccia PF , Pappo AS , Beaupin L , et al. Adolescent and young adult oncology, version 2.2018, NCCN clinical practice guidelines in oncology. J Natl Compr Canc Netw. 2018;16(1):66‐97. doi: 10.6004/jnccn.2018.0001 29295883

[2] National Cancer Institute . Report of the Adolescent and Young Adult Oncology progress Review Group. Closing the gap: research and care imperatives for adolescents and young adults with cancer. 2006.

[3] Smith AW , Seibel NL , Lewis DR , et al. Next steps for adolescent and young adult oncology workshop: an update on progress and recommendations for the future. Cancer. 2016;122(7):988‐999. doi: 10.1002/cncr.29870 26849003 PMC7521143

[4] Nass SJ , Beaupin LK , Demark‐Wahnefried W , et al. Identifying and addressing the needs of adolescents and young adults with cancer: summary of an Institute of Medicine workshop. Oncologist. 2015;20(2):186‐195. doi: 10.1634/theoncologist.2014-0265 25568146 PMC4319626

[5] Sender L , Zabokrtsky KB . Adolescent and young adult patients with cancer: a milieu of unique features. Nat Rev Clin Oncol. 2015;12(8):465‐480. doi: 10.1038/nrclinonc.2015.92 26011488

[6] Tai E , Buchanan N , Townsend J , Fairley T , Moore A , Richardson LC . Health status of adolescent and young adult cancer survivors. Cancer. 2012;118(19):4884‐4891. doi: 10.1002/cncr.27445 22688896 PMC5292773

[7] Chan A , Ng T , Chan R , Poon E , Farid M . Are adolescent and young adult cancer patients affected by ‘chemobrain’?: a call for evidence. Expert Rev Qual Life Cancer Care. 2016;1(3):187‐188.

[8] Phillips‐Salimi CR , Andrykowski MA . Physical and mental health status of female adolescent/young adult survivors of breast and gynecological cancer: a national, population‐based, case‐control study. Support Care Cancer. 2013;21(6):1597‐1604. doi: 10.1007/s00520-012-1701-7 23306935 PMC3644006

[9] Chan A , Poon E , Goh WL , et al. Assessment of psychological distress among Asian adolescents and young adults (AYA) cancer patients using the distress thermometer: a prospective, longitudinal study. Support Care Cancer. 2018;26(9):3257‐3266. doi: 10.1007/s00520-018-4189-y 29644471

[10] Bradford NK , McDonald FEJ , Bibby H , Kok C , Patterson P . Psychological, functional and social outcomes in adolescent and young adult cancer survivors over time: a systematic review of longitudinal studies. Psychooncology. 2022;31(9):1448‐1458. doi: 10.1002/pon.5987 35734846 PMC9544373

[11] Rosgen BK , Moss SJ , Fiest KM , et al. Psychiatric disorder incidence among adolescents and young adults aged 15‐39 with cancer: population‐based cohort. JNCI Cancer Spectrum. 2022;6(6):pkac077. doi: 10.1093/jncics/pkac077 36321955 PMC9733973

[12] Foster M , Niedzwiedz CL . Associations between multimorbidity and depression among breast cancer survivors within the UK Biobank cohort: a cross‐sectional study. BMC Cancer. 2021;21(1):650 . doi: 10.1186/s12885-021-08409-z 34058985 PMC8167936

[13] Ehlers DK , Fanning J , Sunderlage A , Severson J , Kramer AF , McAuley E . Influence of sitting behaviors on sleep disturbance and memory impairment in breast cancer survivors. Cancer Med. 2020;9(10):3417‐3424. doi: 10.1002/cam4.3008 32202706 PMC7221435

[14] Mehnert A , Hartung TJ , Friedrich M , et al. One in two cancer patients is significantly distressed: prevalence and indicators of distress. Psychooncology. 2018;27(1):75‐82. doi: 10.1002/pon.4464 28568377

[15] Molassiotis A , Cheng HL , Lopez V , et al. Are we mis‐estimating chemotherapy‐induced peripheral neuropathy? Analysis of assessment methodologies from a prospective, multinational, longitudinal cohort study of patients receiving neurotoxic chemotherapy. BMC Cancer. 2019;19(1):132. doi: 10.1186/s12885-019-5302-4 30736741 PMC6368751

[16] All of Us Research Program Investigators , Denny JC , Rutter JL , et al. The “All of Us” research program. N Engl J Med. 2019;381(7):668‐676. doi: 10.1056/NEJMsr1809937 31412182 PMC8291101

[17] Hripcsak G , Duke JD , Shah NH , et al. Observational Health Data Sciences and Informatics (OHDSI): opportunities for observational researchers. Stud Health Technol Inform. 2015;216:574‐578. 26262116 PMC4815923

[18] Shi Q , Smith TG , Michonski JD , Stein KD , Kaw C , Cleeland CS . Symptom burden in cancer survivors 1 year after diagnosis: a report from the American Cancer Society's Studies of Cancer Survivors. Cancer. 2011;117(12):2779‐2790. doi: 10.1002/cncr.26146 21495026 PMC3143572

[19] Austin PC . A comparison of 12 algorithms for matching on the propensity score. Stat Med. 2014;33(6):1057‐1069. doi: 10.1002/sim.6004 24123228 PMC4285163

[20] Chao C , Bhatia S , Xu L , et al. Chronic comorbidities among survivors of adolescent and young adult cancer. J Clin Oncol. 2020;38(27):3161‐3174. doi: 10.1200/JCO.20.00722 32673152 PMC7499612

[21] Rassen JA , Shelat AA , Myers J , Glynn RJ , Rothman KJ , Schneeweiss S . One‐to‐many propensity score matching in cohort studies. Pharmacoepidemiol Drug Saf. 2012;21(Suppl 2):69‐80. doi: 10.1002/pds.3263 22552982

[22] All of Us Public Data Browser: Personal and Family Health History.

[23] Austin PC . An introduction to propensity score methods for reducing the effects of confounding in observational studies. Multivariate Behav Res. 2011;46(3):399‐424. doi: 10.1080/00273171.2011.568786 21818162 PMC3144483

[24] Ho D , Imai K , King G , Stuart EA . MatchIt: Nonparametric Preprocessing for Parametric Causal Inference. J Stat Softw. 2011;42(8):1‐28. doi: 10.18637/jss.v042.i08

[25] The R Project for Statistical Computing. https://www.r‐project.org/

[26] Chen DY , Chen CC , Crawford JR , Wang SG . Tumor‐related epilepsy: epidemiology, pathogenesis and management. J Neurooncol. 2018;139(1):13‐21. doi: 10.1007/s11060-018-2862-0 29797181

[27] Myers JS . Proinflammatory cytokines and sickness behavior: implications for depression and cancer‐related symptoms. Oncol Nurs Forum. 2008;35(5):802‐807. doi: 10.1188/08.onf.802-807 18765326

[28] Berger AM , Mooney K , Alvarez‐Perez A , et al. Cancer‐related fatigue, version 2.2015. J Natl Compr Canc Netw. 2015;13(8):1012‐1039. doi: 10.6004/jnccn.2015.0122 26285247 PMC5499710

[29] Thong MSY , van Noorden CJF , Steindorf K , Arndt V . Cancer‐related fatigue: causes and current treatment options. Curr Treat Options Oncol. 2020;21(2):17 . doi: 10.1007/s11864-020-0707-5 32025928 PMC8660748

[30] Chan A , Cheng I , Wang C , et al. Cognitive impairment in adolescent and young adult cancer patients: pre‐treatment findings of a longitudinal study. Cancer Med. 2023;12(4):4821‐4831. doi: 10.1002/cam4.5295 36221816 PMC9972136

[31] Mayo SJ , Lustberg M , Dhillon M , et al. Cancer‐related cognitive impairment in patients with non‐central nervous system malignancies: an overview for oncology providers from the MASCC Neurological Complications Study Group. Support Care Cancer. 2021;29(6):2821‐2840. doi: 10.1007/s00520-020-05860-9 33231809

[32] Chan A , Hertz DL , Morales M , et al. Biological predictors of chemotherapy‐induced peripheral neuropathy (CIPN): MASCC neurological complications working group overview. Support Care Cancer. 2019;27(10):3729‐3737. doi: 10.1007/s00520-019-04987-8 31363906 PMC6728179

[33] Tan CJ , Yip SYC , Chan RJ , Chew L , Chan A . Investigating how cancer‐related symptoms influence work outcomes among cancer survivors: a systematic review. J Cancer Surviv. 2022;16(5):1065‐1078. doi: 10.1007/s11764-021-01097-5 34424498 PMC9489549

[34] Parsons HM , Harlan LC , Lynch CF , et al. Impact of cancer on work and education among adolescent and young adult cancer survivors. J Clin Oncol. 2012;30(19):2393‐2400. doi: 10.1200/JCO.2011.39.6333 22614977 PMC3675694

[35] Lu AD , Zheng Z , Han X , et al. Medical financial hardship in survivors of adolescent and young adult cancer in the United States. J Natl Cancer Inst. 2021;113(8):997‐1004. doi: 10.1093/jnci/djab013 33839786 PMC8328985

[36] Maschio M , Aguglia U , Avanzini G , et al. Management of epilepsy in brain tumors. Neurol Sci. 2019;40(10):2217‐2234. doi: 10.1007/s10072-019-04025-9 31392641

[37] Liang S , Fan X , Zhao M , et al. Clinical practice guidelines for the diagnosis and treatment of adult diffuse glioma‐related epilepsy. Cancer Med. 2019;8(10):4527‐4535. doi: 10.1002/cam4.2362 31240876 PMC6712518

[38] Vecht C , Royer‐Perron L , Houillier C , Huberfeld G . Seizures and anticonvulsants in brain Tumours: frequency, mechanisms and anti‐epileptic management. Curr Pharm des. 2017;23(42):6464‐6487. doi: 10.2174/1381612823666171027130003 29076421

[39] Loprinzi CL , Lacchetti C , Bleeker J , et al. Prevention and management of chemotherapy‐induced peripheral neuropathy in survivors of adult cancers: ASCO guideline update. J Clin Oncol. 2020;38(28):3325‐3348. doi: 10.1200/JCO.20.01399 32663120

