# Supplementary material for: Neuropsychiatric complications and associated management in adolescent and young adult cancer survivors: An All of Us study
Source: Cancer Med. 2023 Oct 30;12(22):20953–63. doi: 10.1002/cam4.6641 (PMC10709746; doi:10.1002/cam4.6641)
Supplement: Supplementary file 1 — Tables S1–S3. [file CAM4-12-20953-s001.docx]

**Supplemental Online Content**

**Neuropsychiatric Complications and Associated Management in Adolescent and Young Adult Cancer Survivors: An *All of Us* Study**

Ivann Agapito^1#^, Ding Quan Ng ^1#^, Joel Milam^2,3^, Argyrios Ziogas^2^, Hoda Anton-Culver^2^, Alexandre Chan^1^

1. School of Pharmacy and Pharmaceutical Sciences, University of California, Irvine, Irvine, CA, USA
2. School of Medicine, University of California, Irvine, Irvine, CA, USA
3. Program in Public Health, University of California, Irvine, Irvine, CA, USA

^#^Co-first authors

***Corresponding author:**

Alexandre Chan, PharmD, MPH, Chair and Professor of Clinical Pharmacy.

Address: 802 W Peltason Dr, Irvine, CA 92697-4625.

Email: [a.chan@uci.edu](mailto:a.chan@uci.edu). Phone: 1-949-824-8896.

**eTable 1. Descriptive statistics of AYAC survivors who answered PMH Surveys and AYAC survivors who did not answer PMH Surveys**

**eTable 2. Prevalence, odds of self-reporting past diagnoses, and age at diagnosis of psychiatric and neurological conditions among AYAC survivors compared to NCMC**

**eTable 3. Proportions and adjusted odds of seeing a provider and taking medications/receiving treatment for neuropsychiatric conditions in AYAC survivors compared to NCMC**

**eTable 1. Descriptive statistics of AYAC survivors who answered PMH Surveys and AYAC survivors who did not answer PMH Surveys**

| **Demographic Variables** | **AYAC^ (N = 788)** | **No PMH Survey AYAC^ (N = 785)** |
| --- | --- | --- |
| **Mean Age when Surveyed (range, SD)** | 41.3 (20 to 75, 9.7) | 39.8 (20 to 73, 9.1) |
| **Mean Years Since First Cancer Diagnosis (range, SD)** | 8.8 (1 to 40, 8.2) | 8.4 (1 to 40, 7.2) |
| **Mean Age at First Cancer Diagnosis (range, SD)** | 32.5 (15 to 39, 5.5) | 31.6 (15 to 39, 5.9) |
| **Gender, n (%)** |  |  |
| Female | 595 (75.5) | 561 (71.5) |
| Male | 182 (23.1) | 221 (28.2) |
| Other** | ≤20 | ≤20 |
| **Sex at Birth, n (%)** |  |  |
| Female | 602 (76.4) | 562 (71.6) |
| Male | 186 (23.6) | 223 (28.4) |
| **Race, n (%)** |  |  |
| White | 693 (87.9) | 536 (68.3) |
| Black or African American | 44 (5.6) | 185 (23.5) |
| Asian | 24 (3.0) | 32 (4.1) |
| More than one population | ≤20 | 18 (2.3) |
| Middle Eastern or North African | ≤20 | ≤20 |
| Native Hawaiian or Other Pacific Islander | ≤20 | ≤20 |
| **Ethnicity, n (%)** |  |  |
| Not Hispanic or Latino | 763 (96.8) | 756 (96.0) |
| Hispanic or Latino | 25 (3.2) | 29 (3.7) |
| **Education, n (%)** |  |  |
| ≤ High School Graduate or GED | 67 (8.5) | 160 (20.4) |
| College One to Three Years | 168 (21.3) | 218 (27.8) |
| College Graduate | 275 (34.9) | 224 (28.5) |
| Advanced Degree | 278 (35.3) | 183 (23.3) |
| **Income, n (%)** |  |  |
| < $10k | 35 (4.4) | 125 (15.9) |
| $10k to $25k | 72 (9.1) | 108 (13.8) |
| $25k to $35k | 48 (6.1) | 50 (6.4) |
| $35k to $50k | 66 (8.4) | 69 (8.8) |
| $50k to $75k | 111 (14.1) | 97 (12.4) |
| $75k to $100k | 105 (13.3) | 91 (11.6) |
| $100k to $150k | 156 (19.8) | 107 (13.6) |
| $150k to $200k | 73 (9.3) | 58 (7.4) |
| > $200k | 122 (15.5) | 80 (10.2) |
| Abbreviations: AYAC - Adolescent and young adult cancer; GED - Tests of General Educational Development; PMH – past medical history; SD - standard deviation. ^ Results reported in compliance with the All of Us Data and Statistics Dissemination Policy prohibiting the display of participant counts ranging 1 to 20.  ** Cumulation of nonbinary and transgender identities. | | |

**eTable 2.** **Prevalence, odds of self-reporting past diagnoses, and age at diagnosis of psychiatric and neurological conditions among AYAC survivors compared to NCMC**

| **Psychiatric Conditions** | **AYAC survivors^** | **1:4 NCMC^** | **P^a^** | **AOR^#^ (95%CI)** | **P^b^** |
| --- | --- | --- | --- | --- | --- |
| **Depression** | 300 (38.1) | 1253 (39.8) | 0.39 | 0.95 (0.81 to 1.14) | 0.653 |
| Child (0-11), N (%) | ≤20 | 64 (5.1) | 0.07 |  |  |
| Adolescent (12-17) | 91 (30.3) | 363 (29.0) | 0.60 |  |  |
| Adult (18-64) | 217 (72.3) | 816 (65.1) | 0.02* |  |  |
| Older adult (65-74) | ≤20 | ≤20 | 0.20 |  |  |
| Elderly (75+) | ≤20 | ≤20 | 0.60 |  |  |
| **Anxiety** | 277 (35.2) | 1100 (34.9) | 0.89 | 1.05 (0.89 to 1.25) | 0.547 |
| Child (0-11) | ≤20 | 56 (5.1) | 0.07 |  |  |
| Adolescent (12-17) | 66 (23.8) | 272 (24.8) | 0.80 |  |  |
| Adult (18-64) | 204 (73.6) | 765 (69.7) | 0.20 |  |  |
| Older adult (65-74) | ≤20 | ≤20 | 0.40 |  |  |
| Elderly (75+) | ≤20 | ≤20 | N/A |  |  |
| **PTSD** | 102 (12.9) | 350 (11.1) | 0.15 | 1.28 (1.01 to 1.63) | 0.045 |
| Child (0-11) | ≤20 | ≤20 | 0.80 |  |  |
| Adolescent (12-17) | ≤20 | 57 (16.3) | 0.20 |  |  |
| Adult (18-64) | 87 (85.3) | 272 (77.7) | 0.10 |  |  |
| Older adult (65-74) | ≤20 | ≤20 | 0.40 |  |  |
| Elderly (75+) | ≤20 | ≤20 | N/A |  |  |
| **ADHD** | 69 (8.2) | 359 (11.4) | 0.034* | 0.79 (0.60 to 1.04) | 0.099 |
| Child (0-11) | ≤20 | 73 (20.4) | 1.00 |  |  |
| Adolescent (12-17) | ≤20 | 69 (19.3) | 0.60 |  |  |
| Adult (18-64) | 40 (58.0) | 215 (60.4) | 0.80 |  |  |
| Older adult (65-74) | ≤20 | ≤20 | 0.70 |  |  |
| Elderly (75+) | ≤20 | ≤20 | N/A |  |  |
| **Bipolar** | 33 (4.2) | 176 (5.6) | 0.12 | 0.81 (0.55 to 1.20) | 0.289 |
| Child (0-11) | ≤20 | ≤20 | 0.20 |  |  |
| Adolescent (12-17) | ≤20 | 53 (30.1) | 0.20 |  |  |
| Adult (18-64) | 27 (81.8) | 112 (63.6) | 0.04* |  |  |
| Older adult (65-74) | ≤20 | ≤20 | N/A |  |  |
| Elderly (75+) | ≤20 | ≤20 | N/A |  |  |
| **Eating Disorder** | 38 (4.8) | 168 (5.3) | 0.57 | 0.92 (0.64 to 1.33) | 0.648 |
| Child (0-11) | ≤20 | ≤20 | 0.80 |  |  |
| Adolescent (12-17) | 25 (65.8) | 86 (51.8) | 0.10 |  |  |
| Adult (18-64) | ≤20 | 73 (44.0) | 0.20 |  |  |
| Older adult (65-74) | ≤20 | ≤20 | N/A |  |  |
| Elderly (75+) | ≤20 | ≤20 | N/A |  |  |
| **Social Phobia** | 29 (3.7) | 105 (3.3) | 0.63 | 1.23 (0.80 to 1.90) | 0.348 |
| Child (0-11) | ≤20 | ≤20 | 0.20 |  |  |
| Adolescent (12-17) | ≤20 | 35 (33.3) | 0.80 |  |  |
| Adult (18-64) | 20 (69.0) | 64 (61.0) | 0.40 |  |  |
| Older adult (65-74) | ≤20 | ≤20 | 0.60 |  |  |
| Elderly (75+) | ≤20 | ≤20 | N/A |  |  |
| **Drug Use Disorder** | ≤20 | 87 (2.8) | 0.35 | 0.81 (0.47 to 1.40) | 0.456 |
| Child (0-11) | ≤20 | ≤20 | N/A |  |  |
| Adolescent (12-17) | ≤20 | ≤20 | 0.70 |  |  |
| Adult (18-64) | ≤20 | 68 (78.2) | 0.70 |  |  |
| Older adult (65-74) | ≤20 | ≤20 | N/A |  |  |
| Elderly (75+) | ≤20 | ≤20 | N/A |  |  |
| **Alcohol Use Disorder** | ≤20 | 107 (3.3) | 0.08 | 0.65 (0.39 to 1.11) | 0.113 |
| Child (0-11) | ≤20 | ≤20 | N/A |  |  |
| Adolescent (12-17) | ≤20 | ≤20 | 0.40 |  |  |
| Adult (18-64) | ≤20 | 89 (83.2) | 0.09 |  |  |
| Older adult (65-74) | ≤20 | ≤20 | 0.50 |  |  |
| Elderly (75+) | ≤20 | ≤20 | N/A |  |  |
| **Personality Disorder** | ≤20 | 65 (2.1) | 0.78 | 1.06 (0.59 to 1.91) | 0.833 |
| Child (0-11) | ≤20 | ≤20 | 0.30 |  |  |
| Adolescent (12-17) | ≤20 | ≤20 | 0.90 |  |  |
| Adult (18-64) | ≤20 | 45 (69.2) | 0.80 |  |  |
| Older adult (65-74) | ≤20 | ≤20 | N/A |  |  |
| Elderly (75+) | ≤20 | ≤20 | N/A |  |  |
| **Autism** | ≤20 | 46 (1.5) | 0.34 | 0.84 (0.38 to 1.84) | 0.655 |
| Child (0-11) | ≤20 | ≤20 | 0.80 |  |  |
| Adolescent (12-17) | ≤20 | ≤20 | 0.70 |  |  |
| Adult (18-64) | ≤20 | 29 (63.0) | 1.00 |  |  |
| Older adult (65-74) | ≤20 | ≤20 | N/A |  |  |
| Elderly (75+) | ≤20 | ≤20 | N/A |  |  |
| **Schizophrenia** | ≤20 | ≤20 | 0.41 | 1.92 (0.63 to 5.79) | 0.249 |
| Child (0-11) | ≤20 | ≤20 | N/A |  |  |
| Adolescent (12-17) | ≤20 | ≤20 | 0.20 |  |  |
| Adult (18-64) | ≤20 | ≤20 | 0.20 |  |  |
| Older adult (65-74) | ≤20 | ≤20 | N/A |  |  |
| Elderly (75+) | ≤20 | ≤20 | N/A |  |  |
| **Neurological Conditions** |  |  |  |  |  |
| **Migraine** | 218 (27.7) | 724 (23.0) | 0.006* | 1.29 (1.08 to 1.55) | 0.006 |
| Child (0-11) | 21 (9.6) | 77 (10.7) | 0.70 |  |  |
| Adolescent (12-17) | 78 (35.8) | 255 (35.3) | 0.90 |  |  |
| Adult (18-64) | 119 (54.6) | 389 (53.9) | 0.80 |  |  |
| Older adult (65-74) | ≤20 | ≤20 | 0.60 |  |  |
| Elderly (75+) | ≤20 | ≤20 | N/A |  |  |
| **Neuropathy** | 109 (13.8) | 142 (4.5) | <0.001* | 3.79 (2.89 to 4.98) | <0.001** |
| Child (0-11) | ≤20 | ≤20 | 0.85 |  |  |
| Adolescent (12-17) | ≤20 | ≤20 | 0.04* |  |  |
| Adult (18-64) | 99 (90.8) | 130 (92.2) | 0.84 |  |  |
| Older adult (65-74) | ≤20 | ≤20 | 0.003* |  |  |
| Elderly (75+) | ≤20 | ≤20 | 0.38 |  |  |
| **Insomnia** | 97 (12.3) | 313 (9.9) | 0.05 | 1.33 (1.04 to 1.71) | 0.024 |
| Child (0-11) | ≤20 | ≤20 | 0.05 |  |  |
| Adolescent (12-17) | 21 (21.6) | 68 (21.8) | 0.99 |  |  |
| Adult (18-64) | 67 (69.1) | 224 (71.8) | 0.64 |  |  |
| Older adult (65-74) | ≤20 | ≤20 | 0.17 |  |  |
| Elderly (75+) | ≤20 | ≤20 | 0.58 |  |  |
| **Chronic Fatigue** | 72 (9.1) | 182 (5.8) | <0.001* | 1.76 (1.31 to 2.35) | <0.001** |
| Child (0-11) | ≤20 | ≤20 | 0.27 |  |  |
| Adolescent (12-17) | ≤20 | 31 (17.0) | 0.37 |  |  |
| Adult (18-64) | 63 (87.5) | 145 (79.7) | 0.14 |  |  |
| Older adult (65-74) | ≤20 | ≤20 | 0.37 |  |  |
| Elderly (75+) | ≤20 | ≤20 | N/A |  |  |
| **Memory Loss** | 53 (6.7) | 87 (2.8) | <0.001* | 2.79 (1.95 to 4.01) | <0.001** |
| Child (0-11) | ≤20 | ≤20 | 0.17 |  |  |
| Adolescent (12-17) | ≤20 | ≤20 | 0.58 |  |  |
| Adult (18-64) | 49 (92.5) | 70 (80.5) | 0.08 |  |  |
| Older adult (65-74) | ≤20 | ≤20 | 0.11 |  |  |
| Elderly (75+) | ≤20 | ≤20 | N/A |  |  |
| **Restless Leg Syndrome** | 55 (7.0) | 154 (4.9) | 0.019* | 1.60 (1.15 to 2.22) | 0.005 |
| Child (0-11) | ≤20 | ≤20 | 0.89 |  |  |
| Adolescent (12-17) | ≤20 | 27 (17.5) | 0.25 |  |  |
| Adult (18-64) | 47 (85.5) | 117 (76.0) | 0.14 |  |  |
| Older adult (65-74) | ≤20 | ≤20 | 0.40 |  |  |
| Elderly (75+) | ≤20 | ≤20 | N/A |  |  |
| **Concussion** | 65 (8.2) | 272 (8.6) | 0.73 | 0.94 (0.71 to 1.25) | 0.661 |
| Child (0-11) | ≤20 | 57 (21.1) | 0.92 |  |  |
| Adolescent (12-17) | ≤20 | 85 (31.5) | 0.58 |  |  |
| Adult (18-64) | 32 (49.2) | 124 (45.9) | 0.60 |  |  |
| Older adult (65-74) | ≤20 | ≤20 | 0.97 |  |  |
| Elderly (75+) | ≤20 | ≤20 | N/A |  |  |
| **Epilepsy** | 44 (5.6) | 77 (2.4) | <0.001* | 2.46 (1.67 to 3.62) | <0.001** |
| Child (0-11) | ≤20 | 21 (27.3) | 0.08 |  |  |
| Adolescent (12-17) | ≤20 | ≤20 | 0.19 |  |  |
| Adult (18-64) | 33 (75.0) | 38 (49.4) | 0.006* |  |  |
| Older adult (65-74) | ≤20 | ≤20 | 0.28 |  |  |
| Elderly (75+) | ≤20 | ≤20 | N/A |  |  |
| **Traumatic Brain Injury** | ≤20 | 52 (1.6) | 0.23 | 1.48 (0.85 to 2.56) | 0.166 |
| Child (0-11) | ≤20 | ≤20 | 0.76 |  |  |
| Adolescent (12-17) | ≤20 | ≤20 | 0.28 |  |  |
| Adult (18-64) | ≤20 | 40 (76.9) | 0.28 |  |  |
| Older adult (65-74) | ≤20 | ≤20 | N/A |  |  |
| Elderly (75+) | ≤20 | ≤20 | N/A |  |  |
| **Spinal Cord Injury** | ≤20 | 48 (1.5) | 0.09 | 1.86 (1.07 to 3.23) | 0.027 |
| Child (0-11) | ≤20 | ≤20 | 0.49 |  |  |
| Adolescent (12-17) | ≤20 | ≤20 | 0.11 |  |  |
| Adult (18-64) | ≤20 | 38 (79.2) | 0.12 |  |  |
| Older adult (65-74) | ≤20 | ≤20 | 0.26 |  |  |
| Elderly (75+) | ≤20 | ≤20 | N/A |  |  |
| **Multiple Sclerosis** | ≤20 | 31 (0.9) | 0.56 | 0.75 (0.73 to 0.77) | 0.53 |
| Child (0-11) | ≤20 | ≤20 | N/A |  |  |
| Adolescent (12-17) | ≤20 | ≤20 | 0.02* |  |  |
| Adult (18-64) | ≤20 | 28 | 0.61 |  |  |
| Older adult (65-74) | ≤20 | ≤20 | N/A |  |  |
| Elderly (75+) | ≤20 | ≤20 | N/A |  |  |
| **Narcolepsy** | ≤20 | ≤20 | 0.32 | 1.61 (0.66 to 3.88) | 0.293 |
| Child (0-11) | ≤20 | ≤20 | N/A |  |  |
| Adolescent (12-17) | ≤20 | ≤20 | 0.47 |  |  |
| Adult (18-64) | ≤20 | ≤20 | 0.47 |  |  |
| Older adult (65-74) | ≤20 | ≤20 | N/A |  |  |
| Elderly (75+) | ≤20 | ≤20 | N/A |  |  |
| **Cerebral Palsy** | ≤20 | ≤20 | 1.00 | 1.02 (0.11 to 9.61) | 0.99 |
| Child (0-11) | ≤20 | ≤20 | N/A |  |  |
| Adolescent (12-17) | ≤20 | ≤20 | N/A |  |  |
| Adult (18-64) | ≤20 | ≤20 | N/A |  |  |
| Older adult (65-74) | ≤20 | ≤20 | N/A |  |  |
| Elderly (75+) | ≤20 | ≤20 | N/A |  |  |
| **Muscular Dystrophy** | ≤20 | ≤20 | 0.57 | 1.60 (0.30 to 8.41) | 0.58 |
| Child (0-11) | ≤20 | ≤20 | 0.15 |  |  |
| Adolescent (12-17) | ≤20 | ≤20 | N/A |  |  |
| Adult (18-64) | ≤20 | ≤20 | 0.15 |  |  |
| Older adult (65-74) | ≤20 | ≤20 | N/A |  |  |
| Elderly (75+) | ≤20 | ≤20 | N/A |  |  |
| **Parkinson's Disease** | ≤20 | ≤20 | 0.84 | 1.64 (0.16 to 16.88) | 0.676 |
| Child (0-11) | ≤20 | ≤20 | N/A |  |  |
| Adolescent (12-17) | ≤20 | ≤20 | N/A |  |  |
| Adult (18-64) | ≤20 | ≤20 | 0.81 |  |  |
| Older adult (65-74) | ≤20 | ≤20 | 0.81 |  |  |
| Elderly (75+) | ≤20 | ≤20 | N/A |  |  |
| **Dementia** | ≤20 | ≤20 | 0.13 | 5.25 (0.67 to 41.08) | 0.11 |
| Child (0-11) | ≤20 | ≤20 | N/A |  |  |
| Adolescent (12-17) | ≤20 | ≤20 | N/A |  |  |
| Adult (18-64) | ≤20 | ≤20 | N/A |  |  |
| Older adult (65-74) | ≤20 | ≤20 | N/A |  |  |
| Elderly (75+) | ≤20 | ≤20 | N/A |  |  |
| Abbreviations: AYAC - Adolescent and young adult cancer; AOR - adjusted odds ratio; CI - confidence interval; NCMC - non-cancer matched controls; P^a^ - p-values for Pearson’s chi-square test or Fisher’s exact test; P^b^ - p-values for multiple logistic regression. ^ Results were reported in compliance with the All of Us Data and Statistics Dissemination Policy prohibiting the display of participant counts ranging 1 to 20. ^#^ Adjusted for sex at birth, race, ethnicity, highest education level, annual household income, and age at survey completion. NCMCs served as the reference group. * P < 0.05 ** Bonferroni-corrected P < 0.00179 for P^b^. | | | | | |

**eTable 3. Proportions and adjusted odds of seeing a provider and taking medications/receiving treatment for neuropsychiatric conditions in AYAC survivors compared to NCMC**

| Neuropsychiatric Condition | Seeing a provider | | | | | Taking medications/receiving treatment | | | | |
| --- | --- | --- | --- | --- | --- | --- | --- | --- | --- | --- |
|  | AYAC (N = 788) | NCMC (N = 3152) | P^a^ Value | AOR^c^ (95%CI) | P^b^ Value | AYAC (N = 788) | NCMC (N = 3152) | P^a^ Value | AOR^c^ (95%CI) | P^b^ Value |
| Neuropathy, n (%) | 61 (7.7) | 112 (3.6) | <0.001* | 2.60 (1.86 to 3.62) | <0.001* | 43 (5.5) | 90 (2.9) | <0.001* | 2.24 (1.53 to 3.28) | <0.001* |
| Chronic fatigue, n (%) | 46 (5.8) | 125 (4.0) | 0.021* | 1.63 (1.14 to 2.33) | 0.007* | 27 (3.4) | 72 (2.3) | 0.067 | 1.64 (1.04 to 2.59) | 0.034* |
| Memory loss, n (%) | 33 (4.2) | 43 (1.4) | <0.001* | 3.41 (2.13 to 5.46) | <0.001* | <20 | 21 (0.7) | <0.001* | 4.17 (2.22 to 7.83) | <0.001 |
| Epilepsy, n (%) | 33 (4.2) | 42 (1.3) | <0.001* | 3.46 (2.15 to 5.58) | <0.001* | 29 (3.7) | 36 (1.1) | <0.001* | 3.48 (2.10 to 5.78) | <0.001* |
| Abbreviations: AYAC - Adolescent and young adult cancer; AOR - adjusted odds ratio; CI - confidence interval; NCMC - Non-cancer matched control; P^a^ - P values for Pearson’s chi-square test; P^b^ - P values for multiple logistic regression.  ^c^ Adjusted for sex at birth, gender, race, ethnicity, highest education level, annual household income, and age at survey completion. NCMCs served as the reference group. *P < 0.05 | | | | | | | | | | |
